# Respiratory Sinus Arrhythmia as a Therapy Measurement Strategy: The Example of Functional Analytic Psychotherapy

**DOI:** 10.53520/rdts2023.10557

**Published:** 2023

**Authors:** Daniel W.M. Maitland, Chung Xiann Lim, William H. O’Brien, Jacob A. Lewis, Cambria L. Davis

**Affiliations:** 1 Bowling Green State University, OH, USA; 2 University of Louisville, KY, USA

**Keywords:** Therapy outcome, Biophysiological measurements, Polyvagal theory

## Abstract

**Introduction:**

Respiratory sinus arrhythmia (RSA) may be a psychophysiological measure that can gauge change processes in psychotherapy. RSA is a cardiovascular marker of nervous system functioning and has been proposed as an index of social engagement. Given the emphasis on social relationships in Functional Analytic Psychotherapy (FAP), RSA may be useful for understanding the process of change in FAP. The current study details the logic of using RSA as a measurement tool and provides data to establish proof of concept.

**Methods:**

RSA data were collected from three participants in a multiple baseline experiment which involved a control condition of reading two-person plays and a FAP condition. Sessions were coded using the FAP Rating Scale. RSA was calculated for each session and each client-therapist interaction.

**Results:**

RSA higher during FAP sessions (P1Mean = 6.60, P2Mean = 6.41, P3Mean = 6.32) than the control condition (P1Mean = 6.29, P2Mean = 5.79, P3Mean = 5.96).

**Conclusions:**

Data are consistent with outcomes that might be anticipated during therapy and suggest RSA could improve as a function of treatment. RSA was able to be isolated for therapeutic moves when coded at the turn-by-turn level. Thus, data suggest RSA’s utility for measuring therapy processes and outcomes.

## Introduction

Heart rate variability, frequently indexed as respiratory sinus arrhythmia (RSA), is thought to measure perceptions of social threat and safety^1^. RSA is an index of systematic changes in the interbeat interval that corresponds to breathing. During inhalation, the interbeat interval typically shortens and vice versa. When plotted across time, this pattern of variation in heart rate forms a sine wave. If the amplitude of the sine wave increases heart rate variability will invariably increase as well. Thus, RSA can be used as a measure of heart rate variability.^2^ RSA can also provide insight into vagal activity as well as the polyvagal and neurovisceral central integration systems that regulate the social-emotional context of psychotherapy^3^.

RSA could be a biomarker of engagement in the behaviors that make up the social intimacy process^4^. Higher RSA has been associated with behaviors that could be conceptualized as part of the interpersonal intimacy process^5^. The association between RSA and client experiences should be particularly strong in Functional Analytic Psychotherapy (FAP) given that it is thought to inherently create social intimacy^6^. FAP, is grounded on principals of operant conditioning, specifically on the use of therapist personal responses to the client to differentially reinforce desirable behaviors in-session while putting problematic behaviors on extinction. These behaviors, termed clinically relevant behaviors (CRB), are the focal point of therapy. Thus, FAP therapists use the relationship between them and the client to change the behaviors interfering with the client’s daily life. Researchers have found that almost all clinically relevant behaviors can be mapped onto the interpersonal model of intimacy, leading to a dissemination and implementation strategy of FAP termed the Awareness, Courage, and Love Model (ACL)^7^. In the ACL model, instead of idiographically defined targets, key aspects of relationship building behaviors are treated as CRBs. Deficits in these behaviors result in disruptions to the intimacy process, impacting the ability to form connections with others. This conceptualization of FAP is substantiated by findings that have shown that FAP is an effective intervention for reducing fear of intimacy^8^ and strengthening the therapeutic alliance presumably through the intimacy process^9^. The present manuscript presents proof of concept for the use of RSA measurement as a therapy process or outcome measure in FAP^10^.

## Scientific Methods

### Participants

To be included in the study, participants had to be between the ages of 18 and 35, speak English fluently, score one standard deviation below the mean on the Fear of Intimacy Scale^11^ (FIS) or the UCLA Loneliness scale^12^, measures thought to be impacted by the intimacy process. Participants had to meet diagnostic criteria for major depressive disorder, social phobia, generalized anxiety disorder, or PTSD which are viewed as prototypical intervention targets for FAP and are thus ideal for efficacy research. Individuals were not eligible to participate in the study if they met diagnostic criteria for eating disorders or alcohol/drug abuse or dependence, reported acute suicidal ideation, scored above a 30 on the psychopathy checklist^13^, reported cognitive impairment, were receiving concurrent therapy or had changed their use of psychotropic medications in the last 8 weeks.

Participant 1 was a 19-year-old woman who identified as White and Latina. Participant 1 endorsed a significant number of depression, PTSD, and panic symptoms. Participant one struggled with any amount of self-disclosure, frequently talking about the context of their life without any mention of themselves in the narrative. Thus, treatment for Participant 1 focused on talking about herself in any capacity. She dropped out of the study after seven sessions without contacting the therapist. Participant 2 was a 19-year-old White woman who did not endorse any ethnic identities. She reported a significant number of depression, PTSD, and social anxiety symptoms. Participant 2’s treatment had an emphasis on disclosure such as using I statements instead of deflecting self-disclosure. Participant 3 was also a 19-year-old White woman who did not report any ethnic identity. She endorsed a high number of depression and generalized anxiety symptoms. Participant 3 reported that she wanted to work on making emotional disclosures and letting others show care for her.

### Protocol

The baseline (control) condition of the current study involved the participant and therapist reading out loud lines from two-person plays together. Each individual took the role of one of the characters in the play and the study therapist read any stage instructions. Participants stayed in this condition for a minimum of three sessions until stability was demonstrated on the FIS, UCLA, or on RSA as evidenced by no meaningful change in scores or scores moving in the undesired direction for three sessions in a row. Only one participant per week transitioned to the therapy condition. In the therapy condition, the therapist administered a FAP condition guided by the ACL.

The FAP Rating Scale^14^ (FAPRS) is a coding system designed to assess the implementation of FAP at a turn-by-turn level. A single code is applied to each client or therapist turn of speech. The coding system details client CRB1, CRB2, and CRB3 as well as therapist effective responding to these behaviors (TRB1, TRB2, TRB3). The coding system also details discussion of CRB1 and CRB2 occurring outside of session (O1 & O2) and therapist responding to those discussions (RO1 & RO2). There are codes for progressing therapy by the client (CPR) and therapist (TPR) and codes for focusing on the relationship between the client (CTR) and therapist (TTR). Finally, there is a code for therapists evoking of CRB (ERB). A designation of NC is used when no code is appropriate.

The therapist was the first author of the current study. Study assessors were M.A. students who operated independently from the study therapist. Coders for the current study were two Ph.D.-level clinical psychologists. All coding was carried out independently apart from one session which was coded in conjunction with the study therapist for training purposes.

ECG electrodes were placed on the participants using a Lead II configuration. ECG signals were corrected for artifacts using MindWare technologies software. High Frequency HRV was calculated by extracting the percentage (or power) of the ECG signal that fell into a frequency range of .12 - .4Hz during each session and every turn of speech. This was then converted to RSA.

### Statistical Analysis

Consistent with extant literature on multiple baseline designs, intervention effects on RSA were evaluated using visual inspection. To explore the association between therapy behavior and RSA, the average RSA was calculated for each turn of speech. The original data set of RSA data contained 3,248 different observations including 845 observations from Participant 1, 1,299 observations from Participant 2, and 1,103 observations from Participant 3 after outliers were removed. RSA was calculated for each turn of speech based on FAPRS coding and calculated to reflect the RSA over the course of the entire session.

FAPRS codes were determined by two raters. When both raters agreed, the specific codes were used. If the two raters disagreed, the first authors reviewed the codes proposed by raters and selected the best fitting code of the two. Coders agreed on 78.61% of codes and had a kappa score of ϰ = .832, p < .001).

## Results

**Figure 1.**
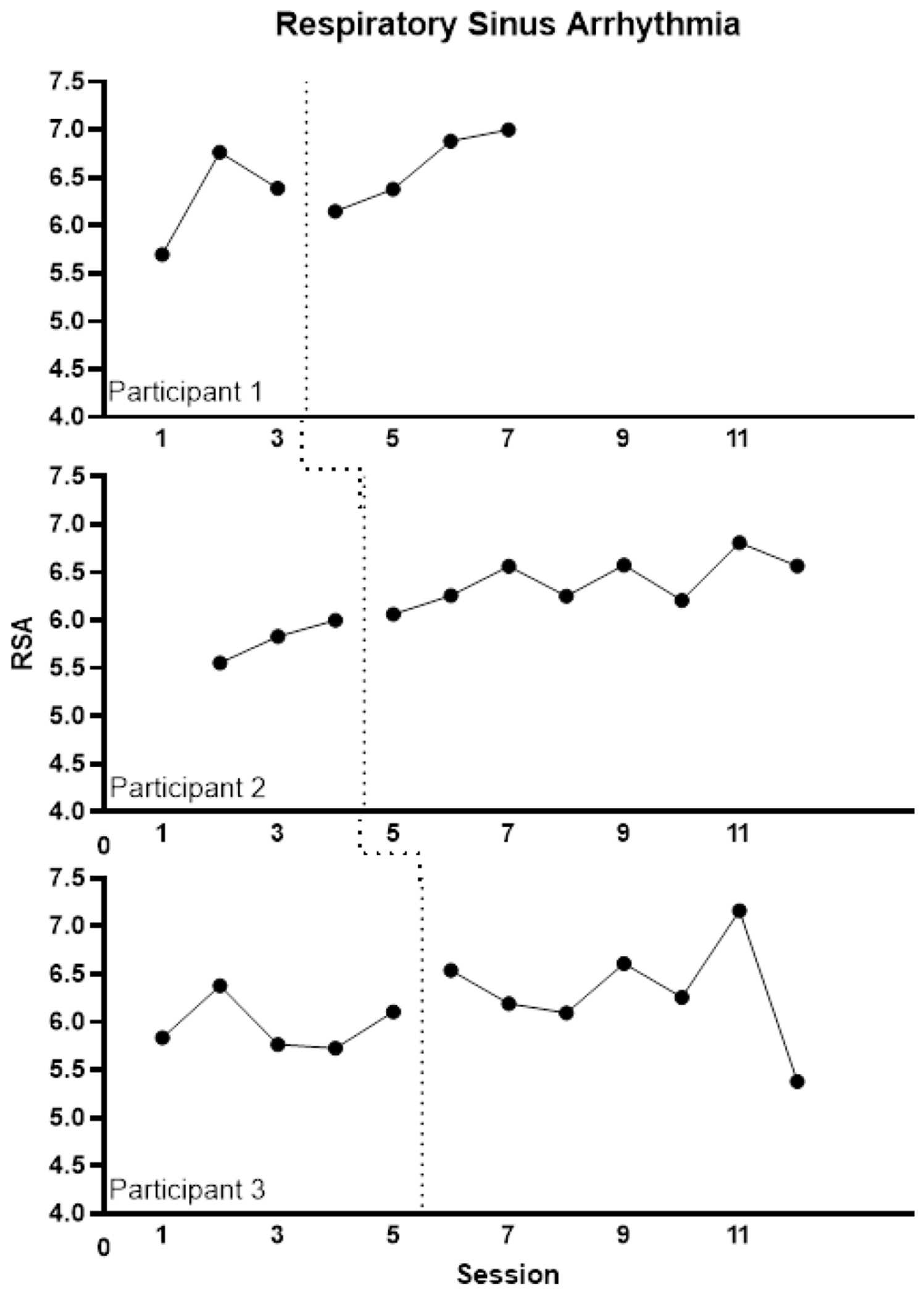
Individual Participant Charts of Respiratory Sinus Arrhythmia (RSA)

Visual analyses of participants’ average RSA during each session (Figure 1) were inconclusive. Participant 1’s data had a general upward trend with a drop at the start of the FAP condition. Participant 2’s data showed no deviations from the trend established during baseline in the FAP condition. However, there was no overlapping data for Participant 2 between the phases suggesting an effect due to transitioning to treatment. Participant 3’s data had a level shift following the transition to the FAP phase with a notable decrease in RSA during the final session. Variability in baseline data for Participant 3 led to a significant overlap of data between phases, limiting interpretability. Table 1 presents the average RSA and standard deviation during each turn of speech by code for each participant.

## Discussion

During the control condition, RSA was typically lower indicating fewer feelings of safety and less social engagement during the play reading condition. This suggests a differential effect on RSA as a function of therapy. When RSA was lower during treatment than in the control condition, indicating that therapy felt more threatening, it was either at the start or end of therapy. It is possible that the individuals in the study felt threatened when they started talking about themselves more. Similarly, ending relationships can make individuals feel unsafe, especially when it involves discussing the full extent of feelings experienced in that relationship, as is typically done in FAP. This is also arguably the goal of treatment and is indicative of therapeutic growth as a function of treatment. When RSA is viewed as an outcome measure, data suggest that the treatment was beneficial, perhaps with the exception of Participant 3, but it is unclear if the results can be attributed to therapy given a general trend increase in RSA over time.

The study design prevented meaningful use of RSA as a process variable, however, capturing RSA at the turn-by-turn level may facilitate research into the effectiveness of therapeutic moves in FAP. Traditionally, when FAP has been analyzed at the turn-by-turn level^15^, researchers have focused on the count of a specific type of therapeutic move or the timing of those moves. Despite such studies having a meaningful impact on understanding the mechanism of action in therapy, they are limited in what they can contribute to understanding effective therapeutic moves. In contrast, by measuring RSA, researchers could track the same behavior over time and assess if the same behavior feels safer over time. Similarly, researchers could engage in more complex analyses to predict therapeutic outcomes by obtaining a quantitative value for data that has historically been exclusively categorical.

Findings must be interpreted with caution. Findings linking RSA to turns of speech are focused on how RSA applies to FAP as a treatment rather than any self-reported feelings of the participant. Additionally, the turns of speech during therapy were very short. It is possible that these short observation periods impacted the validity of the data. Similarly, like all single subject design studies, especially with a participant who withdrew from the study, the small sample size limits the generalizability of the findings. Additionally, the nature of the study limited the ability to conduct meaningful statistical interpretations of the data. Future researchers may consider using FAP analog studies where researchers could experimentally manipulate vulnerability and responsiveness. Such a study could allow for comparisons of different responses to participant behavior and would allow for more stringent controls of RSA.

## Conclusions

Capturing RSA during therapy may lead to unique analyses of the process and efficacy of treatment. FAP naturally lends itself to this measurement technique due to a focus on social connection. The current study demonstrates the feasibility and techniques needed to use RSA as a treatment process and outcome measure in FAP.

## Figures and Tables

**Table 1.** Average RSA and standard deviation for specific FAPRS code for each participant.

Code		Participant		

	1	2	3	Note
**CPR**	6.31 ± 1.17	5.97 ± 1.09	6.15 ± 1.04	Client positive session progression
**CRB1**	5.89 ± 1.68	6.18 ± 0.98	6.96 ± 1.07	In-session problem
**CRB2**	6.48 ± 1.12	5.99 ± 0.90	6.83 ± 1.19	In-session improvement
**CRB3**	6.73 ± 0.84	6.17 ± 1.07	6.46 ± 1.02	Client descriptions of important functional relationship
**CTR**	5.78 ± 1.26	5.99 ± 1.24	5.71 ± 0.97	Focus on therapeutic relationship
**ERB**	6.40 ± 0.92	6.68 ± 1.04	6.78 ± 0.91	Therapist evokes CRB
**NC**	7.00 ± 1.23	6.39 ± 0.89	5.64 ± 0.80	No code
**O1**	5.83 ± 2.10	5.15 ± 0.71	6.38 ± 0.83	Outside problems
**O2**	6.15 ± 1.04	6.05 ± 1.13	6.32 ± 1.29	Outside improvements
**RO1**	5.22 ± 0.86	6.09 ± 0.09	7.01 ± 0.56	Therapist responds to O1
**RO2**	5.77 ± 1.48	6.17 ± 2.13	5.99 ± 0.84	Therapist responds to O2
**TPR**	6.59 ± 1.23	6.37 ± 1.05	6.17 ± 1.03	Therapist positive session progression
**TRB1**	6.60 ± 1.35	6.37 ± 1.08	7.01 ± 0.47	Therapist shapes in-session problem
**TRB2**	6.28 ± 1.56	6.99 ± 1.25	6.40 ± 0.92	Therapist shapes in-session improvement
**TRB3**	6.91 ± 1.17	6.97 ± 0.95	6.42 ± 1.15	Therapist shapes description of functional relationships
**TTR**	6.87 ± 1.20	6.69 ± 1.01	6.53 ± 0.96	Therapist focuses on therapeutic relationship

Overall	6.51 ± 1.49	6.29 ± 1.45	6.24 ± 1.30	
